# Plasmacytoid Dendritic Cells Play a Role for Effective Innate Immune Responses during *Chlamydia pneumoniae* Infection in Mice

**DOI:** 10.1371/journal.pone.0048655

**Published:** 2012-10-31

**Authors:** Timothy R. Crother, Jun Ma, Madhulika Jupelli, Norika Chiba, Shuang Chen, Anatoly Slepenkin, Randa Alsabeh, Ellena Peterson, Kenichi Shimada, Moshe Arditi

**Affiliations:** 1 Pediatrics Infectious Diseases, Cedars-Sinai Medical Center, University of California Los Angeles, Los Angeles, California, United States of America; 2 Inflammatory Bowel and Immunobiology Research Institute, Cedars-Sinai Medical Center, Los Angeles, California, United States of America; 3 Department of Pathology, University of California Irvine, Irvine, California, United States of America; 4 Department of Pathology and Laboratory Medicine, Cedars-Sinai Medical Center, University of California Los Angeles, Los Angeles, California, United States of America; National University, Costa Rica

## Abstract

Plasmacytoid dendritic cells (pDCs) are known for their robust antiviral response and their pro-tolerance effects towards allergic diseases and tissue engraftments. However, little is known about the role pDCs may play during a bacterial infection, including pulmonary *Chlamydia pneumoniae* (CP). In this study, we investigated the role of pDCs during pulmonary CP infection. Our results revealed that depletion of pDCs during acute CP infection in mice results in delayed and reduced lung inflammation, with an early delay in cellular recruitment and significant reduction in early cytokine production in the lungs. This was followed by impaired and delayed bacterial clearance from the lungs which then resulted in a severe and prolonged chronic inflammation and iBALT like structures containing large numbers of B and T cells in these animals. We also observed that increasing the pDC numbers in the lung by FLT3L treatment experimentally results in greater lung inflammation during acute CP infection. In contrast to these results, restimulation of T-cells in the draining lymph nodes of pDC-depleted mice induced greater amounts of proinflammatory cytokines than we observed in control mice. These results suggest that pDCs in the lung may provide critical proinflammatory innate immune responses in response to CP infection, but are suppressive towards adaptive immune responses in the lymph node. Thus pDCs in the lung and the draining lymph node appear to have different roles and phenotypes during acute CP infection and may play a role in host immune responses.

## Introduction

*Chlamydia pneumoniae* (CP), an obligate intracellular pathogen, is a common respiratory pathogen that causes atypical pneumonia and is linked to chronic inflammatory diseases such as atherosclerosis and asthma [1]–[4]. Due to this link with important chronic inflammatory diseases, and that fact that most people will become seropositive for CP infection eventually [5], understanding the nature of CP infections and how the immune system responds to it is of great importance to dissect its potential role in these chronic inflammartory diseases. Innate immune responses to CP infection are driven by pattern recognition receptors (PRRs) such as TLR2, TLR4, and the Nod/Rip2 signaling cascade [6]–[8]. Additionally, activation of the NLRP3 inflammasome and IL-1β signaling critically direct proper immune responses to CP infection [9]. Alveolar macrophages and airway epithelial cells are the first cells that will detect and respond to CP lung infection. They, along with conventional dendritic cells (cDCs) and the rapid influx of neutrophils, are thought in direct the appropriate immune responses to CP infection. More recently, CP infection was shown to induce an influx of pDCs into the lungs of mice [10], [11].

Plasmacytoid dendritic cells (pDCs) are well known for their ability to make large amounts of type I Interferon (IFN) in response to viral infections [12]. Additionally, pDCs play an important immunosuppressive role by modulating Treg activity and preventing allergic sensitizations [10], [13], [14]. More recently, pDCs were implicated in the development of central tolerance in the thymus [15]. However, very little is known about the role of pDCs during bacterial infections, including CP. In a brief report, Ang *et al* found that pDCs play a role in controlling *Legionella pneumophila* infection, and that this was independent of type I IFN production [16]. In another study, Takagi *et al* found that depletion of pDCs resulted in decreased inflammation, enhanced clearance, and reduced mortality during *Listeria monocytogenes* infection [17]. However, Type I IFN receptor deficient mice (IFNAR-/-) display enhanced clearance of *L. monocytogenes*, indicating again that Type I IFN is not always the primary role for pDCs, or even beneficial, during bacterial infection [18].

In this study, we sought to understand the potential role pDCs might play during a pulmonary bacterial infection. In order to do this, we depleted pDCs during CP infection, an important human pathogen, and found defective innate immune responses early on. Reduced inflammation and cytokine production 3 days after infection resulted in delayed CP clearance and this led to a secondary and persistent chronic inflammation by day 21 that was characterized by excess IL-12 production and increases in CD8+ T-cells, B-cells, and conventional dendritic cells (cDC). Increasing the numbers of pDCs by FLT3L treatment experimentally in our model led to greater lung inflammation in response to CP infection. Finally, although pDC depletion resulted in defective initial innate immune responses in the lung against CP infection, pDC depletion in the draining lymph nodes (DLN) resulted in increased T-cell responses, indicating different roles and phenotypes for pDCs between the lung and the mediastinal LN.

## Results

### pDCs are required for proper innate immune responses to Chlamydia pneumoniae infection in mice

**Figure 1:**
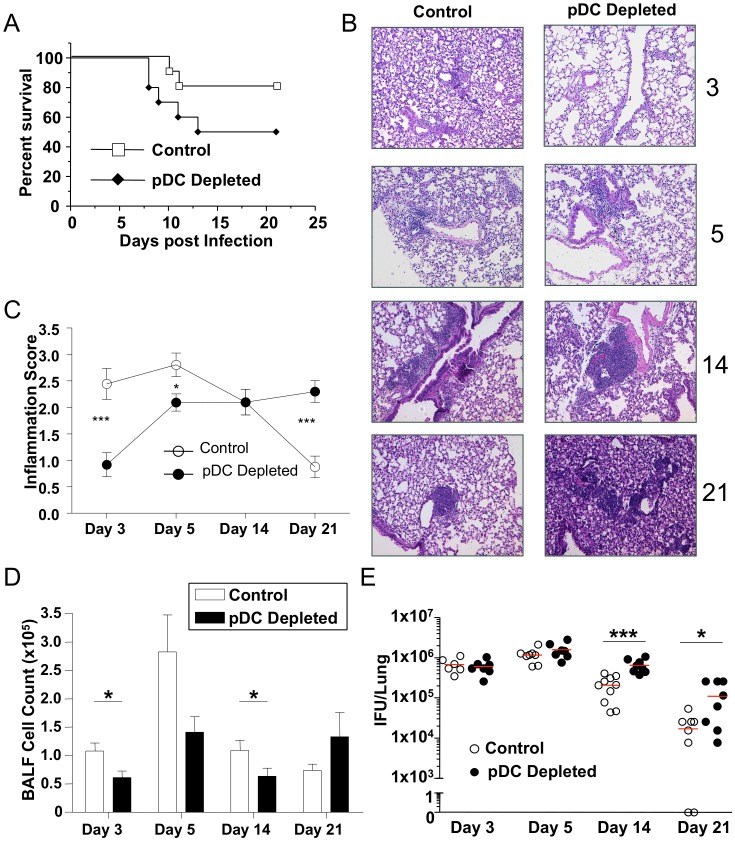
pDCs are required for proper inflammatory responses during *Chlamydia pneumoniae* infection in mice. (**A**) C57Bl/6 mice were infected with 3.5×10^6^ IFU CP i. t. Mice were injected i. p. with either 500 µg mAb 927 or IgG control (n = 10) every other day starting at day -1 and survival was assessed. (**B–E**) C57Bl/6 mice were infected with 1×10^6^ IFU CP i. t. and were injected i. p. with either 500 µg mAb 927 or IgG control (n = 7–10) every other day. Mice were sacrificed on days 3, 5, 14, and 21 after infection. Lungs were analyzed by (**B**) H&E staining (100x), (**C**) inflammation score, (**D**) BALF cell count, and (**E**) bacterial burden. Data for all experiments shown represent at least two independent experiments (pooled together). *p<0.05, **p<0.01, ***p<0.001 (Student's *t* test used unless otherwise noted).

It was previously reported that pDCs accumulate in the lungs of mice infected with CP [10]. In order to investigate the role of pDCs during CP infection in mice, we utilized an antibody depletion model using a monoclonal antibody to the pDC surface protein BST2 [19]. We first investigated the effect of pDC ablation on CP infection induced mouse mortality model and observed a trend towards increased mortality with pDC depletion (Figure 1A). We reduced the dose of CP infection from 3.5×10^6^ IFU to 1×10^6^ IFU to study the effects of pDC depletion on the immune response (Figure S1A and B). Mice were sacrificed at days 3, 5, 14, and 21 after infection. H&E staining revealed a delay in lung inflammation on days 3 and 5 post infection, followed by a significant increase in inflammation 21 days after infection (Figure 1B and C). This correlated with decreases in total Bronchoalveolar Lavage Fluid (BALF) cell counts days 3–14 post infection and trend towards an increase in BALF cell counts at day 21 (Figure 1D). Additionally, bacterial clearance in the lung was significantly delayed by days 14 and 21 during pDC depletion (Figure 1E).

**Figure 2:**
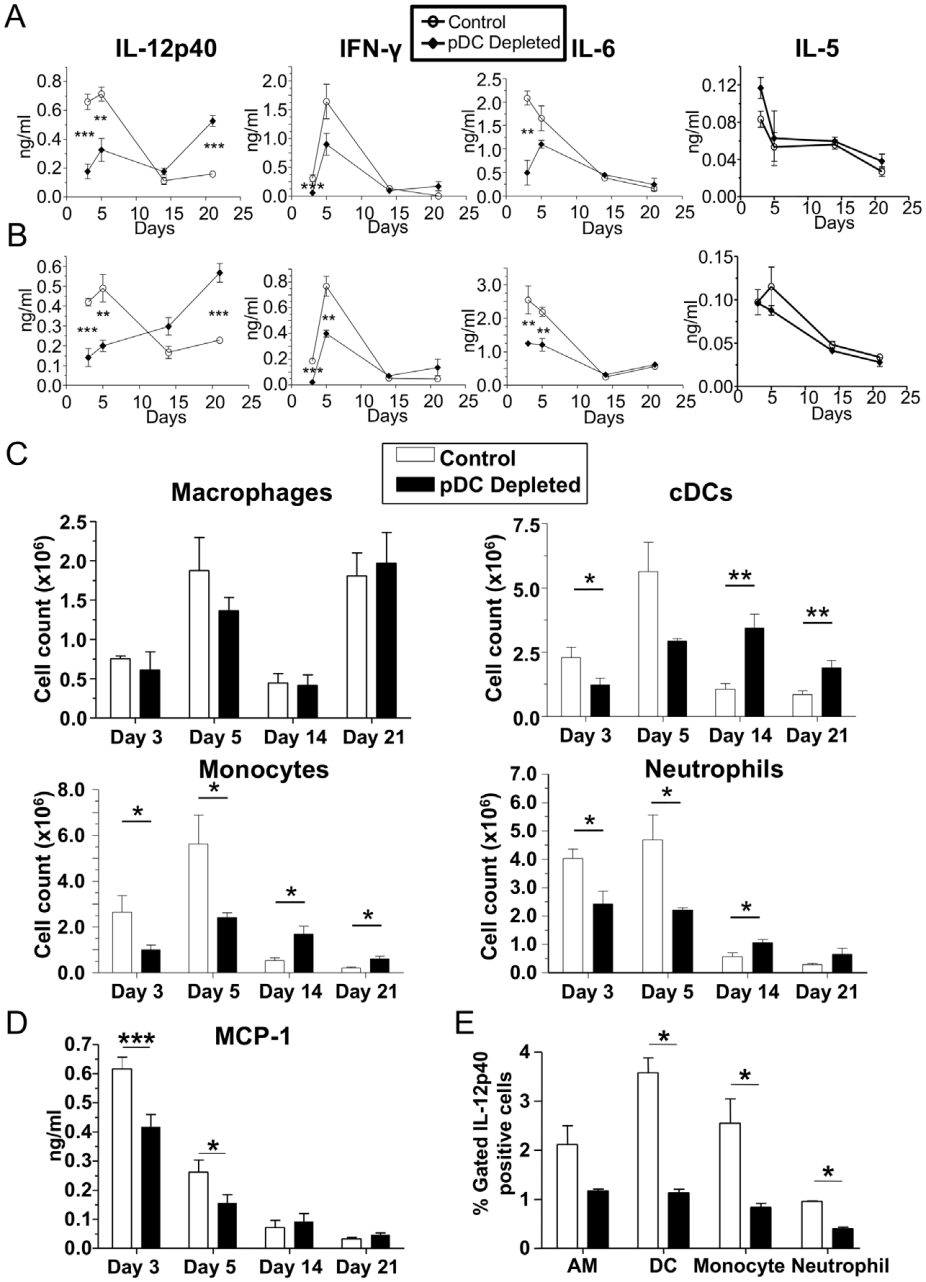
pDC depletion during CP infection results in decreased cytokine production and delayed cellular infiltration. (**A–E**) C57Bl/6 mice were infected with 1×10^6^ IFU CP i. t. and were injected i. p. with either 500 µg mAb 927 or IgG control (n = 7–10) every other day. Mice were sacrificed on days 3, 5, 14, and 21 after infection. Cytokines (IL-12p40, IFN-γ, IL-6, and IL-5) were assessed by ELISA in the (**A**) BALF and (**B**) lung homogenates. (**C**) Alveolar macrophages, cDCs, monocytes, and neutrophils were counted in lung single cell suspensions by Flow cytometry. (**D**) MCP-1 was measured in lung homogenates by ELISA. (**E**) Intracellular IL-12p40 expression was measured in various immune cells by Flow cytometry. Data for all experiments shown represent at least two independent experiments (pooled together). *p<0.05, **p<0.01, ***p<0.001 (Student's *t* test used unless otherwise noted).

We next investigated cytokine levels in lung homogenates and the BALF. In pDC-depleted mice, IL-12p40, IFN-γ, and IL-6 were all reduced at days 3 and 5 after infection compared with control animals (Figure 2A and B). These data correlated with the initially reduced inflammation seen at days 3 and 5 after infection (Figure 1C). However, while IFN-γ and IL-6 were present at similar concentrations compared with control mice at days 14 and 21 post infection, IL-12p40 was significantly increased at day 21 in pDC-depleted animals (Figure 2A and B). This late increase in IL-12p40 correlated with the secondary increase in lung inflammation that we observed at day 21 shown in Figure 1C. Importantly, IL-5 levels did not differ at any time point between pDC-depleted animals or controls (Figure 2A and B).

When we analyzed the inflammatory cells in the lung by Flow cytometry, we found that while alveolar macrophages (AM) numbers did not change in pDC-depleted mice, neutrophils, monocytes, and cDC numbers were reduced early in these mice when compared with control animals (Figure 2C). However, by days 14 and 21 after infection, pDC-depleted animals had increased numbers of cDCs, neutrophils, and monocytes in the lung compared to control animals. Since we saw a reduction in infiltrating monocytes in pDC-depleted animals at day 3 and 5 post infection, we investigated the concentration of the chemokine MCP-1 in lung homogenates and found that indeed MCP-1 concentrations were significantly reduced in these animals early on at 3 and 5 days after infection (Figure 2D). While pDC depletion did not affect lung inflammation in uninfected mice (Figure S2A, B, E), there were some minor changes in cytokine amounts in the BALF and lung homogenates (Figure S2C, D). However, cytokine amounts in naïve lungs were generally 1 log lower compared with infected animals.

IL-12 is in an important T_h_1 skewing cytokine that also affects IFN-γ production and is critical during *C. pneumonaie* infections [20]. Therefore we next investigated the reduction of IL-12p40 three days after infection by assessing the numbers of IL-12p40 producing cells by intracellular Flow cytometry. We found that the percentage of IL-12p40 positive staining cells was significantly reduced in multiple cell types, including alveolar macrophages, cDCs, monocytes, and neutrophils (Figure 2E). This data indicate that the reduction in IL-12p40 early during infection in pDC-depleted animals is not simply due to reduced numbers of monocytes and neutrophils, as AM and cDC also had fewer numbers of IL-12p40 producing cells, yet their percentages were either increased or did not change during those time points.

### pDCs in the Draining Lymph Node are Suppressive During CP Infection

**Figure 3:**
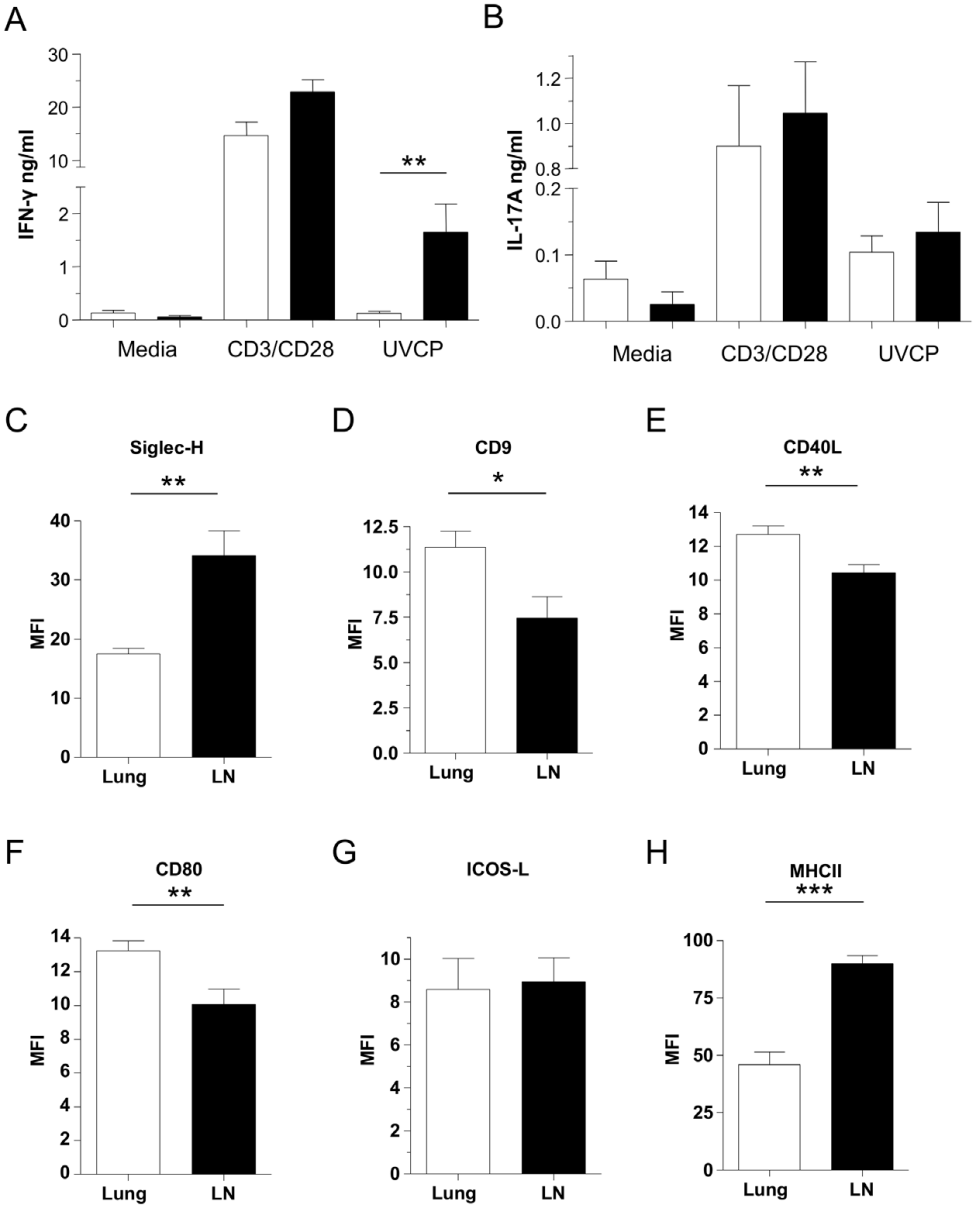
pDCs have a suppressive phenotype in the draining lymph node during CP infection. (**A–B**) C57Bl/6 mice were infected with 1×10^6^ IFU CP i. t. and were injected i. p. with either 500 µg mAb 927 or IgG control (n = 6) every other day. Mice were sacrificed 5 days after infection. Mediastinal lymph nodes were harvested and single cells were stimulated with either CD3/CD28 antibodies or UV killed CP for three days. (**A**) IFN-γ and (**B**) IL-17A production was measured in the supernatants by ELISA. **p<0.01, (Mann-Whitney) (**C–H**) C57Bl/6 mice were infected with 1×10^6^ IFU CP i. t. (n = 9–10) and were sacrificed 5 days after infection. Mediastinal lymph nodes were harvested and pDC surface markers were assessed for by Flow cytometry: (**C**) Siglec-H, (**D**) CD9, (**E**) CD40L, (**F**) CD80, (**G**) ICOS-L, and (**H**) MHCII. Data for all experiments shown represent at least two independent experiments pooled together. *p<0.05, **p<0.01, ***p<0.001 (Student's *t* test used unless otherwise noted).

After we established that the innate immune responses in the lung appeared to be defective in pDC-depleted mice, we next investigated the effect of pDC depletion in the mediastinal lymph node 5 days after CP infection. Total draining lymph node (DLN) cells were stimulated with CD3/CD28 antibodies, or restimulated with UV killed CP (UVCP) and the supernatants were assayed for cytokine production. To our surprise, IFN-γ production was significantly increased in pDC-depleted animals compared to controls (Figure 3A), despite the initial defective innate immune responses found in the lung. This was true for UVCP restimulation and trended towards an increase with CD3/CD28 stimulation. However, IL-17A production was not altered between the two groups (Figure 3B). We examined the cell populations in the DLN and found that both groups had similar numbers of cells (Figure S3A). However, while cDC numbers did not change in the pDC-depleted mice (Figure S3B), these animals actually had reduced numbers of CD4+ and CD8+ T-cells (Figure S3C and D). Finally, the percentages of Tregs and B-cells were unaltered between the pDC-depleted or undepleted control groups (Figure S3E and F).

Since the DLN in pDC-depleted animals actually had reduced T-cell levels compared with controls, yet produced more IFN-γ, we next compared the surface markers of the DLN pDCs with the lung pDCs to determine if the two populations differed. In support of our restimulation experimental data, the DLN pDCs expressed increased levels of Siglec-H on their surface, and decreased levels of CD9, CD40L, and CD80, all indicative of a suppressive phenotype (Figure 3C–F) [21]. We did not find any difference in ICOSL expression levels and to our surprise, we found increased levels of MHCII on the surface of DLN pDCs (Figure 3G and H). These data strongly suggest that pDCs in the DLN have acquired a suppressive phenotype (compared to the pDCs in the lung) and their depletion results in increased IFN-γ production during CP infection after stimulation.

### Diphtheria toxin induced ablation of pDCs and CP infection

**Figure 4:**
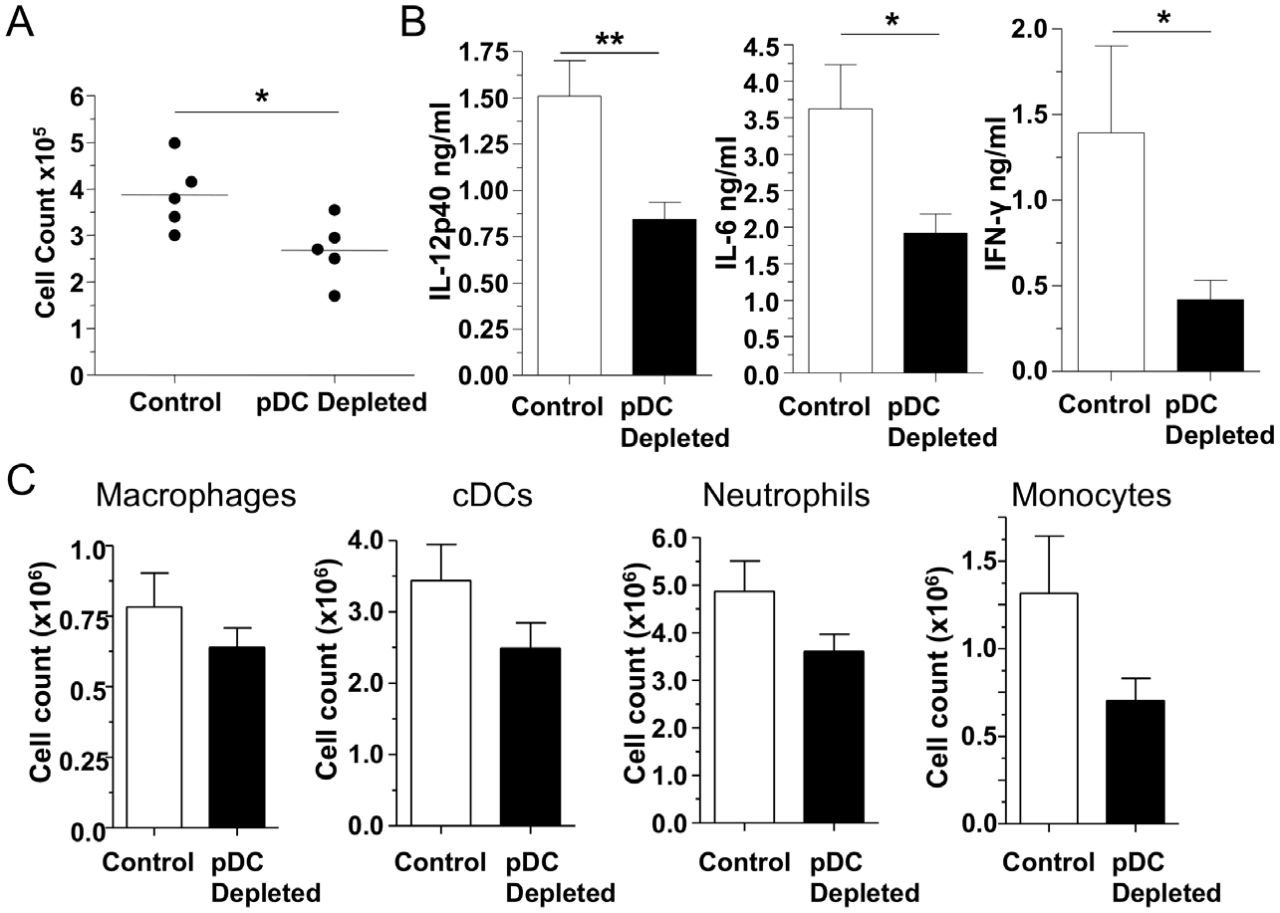
pDC depletion using the diphtheria toxin ablation model has reduced cytokine levels in the BALF. BDCA2-DTRtg mice were infected with 1×10^6^ IFU CP i. t. and were injected i. p. with either 100 ng DT or PBS control (n = 5) every other day. Mice were sacrificed 3 days after infection. (A) BALF cell count (Mann-Whitney). (**B**) IL-12p40, IL-6, and IFNγ concentrations were measured in the BALF by ELISA. (**C**) Alveolar macrophages, cDCs, monocytes, and neutrophils were counted in lung single cell suspensions by Flow cytometry (Mann-Whitney). Data for all experiments shown represent at least two independent experiments pooled together. *p<0.05, **p<0.01, ***p<0.001 (Student's *t* test used unless otherwise noted).

While the antibody we used predominately depletes pDCs, one potential problem with the antibody depletion model of pDCs (using BST2 epitopes) is that BST2 may become upregulated on other immune cells during inflammation which could potentially confound the data by depleting other cell types besides pDCs [19]. Thus in order to confirm that our observations were due to pDC depletion and not the depletion of other cell types as well, we used BDCA2-DTRtg mice where pDCs can be specifically ablated after administration of diphtheria toxin (DT) [22]. Mice were infected as before and pDCs were depleted by the administration of DT (Figure S4A). We looked at the earliest time point where we found differences before (day 3) and found that pDC-depleted animals in this model also had a reduced BALF cell count (Figure 4A) compared with control animals, similar to the antibody depletion model (Figure 1D). Additionally we found significantly reduced concentrations of IL-12p40, IL-6, and IFNγ in the BALF (Figure 4B). There was also a strong trend towards reduced cell numbers, including cDCs, neutrophils, and monocytes in the lungs of pDC-depleted mice (Figure 4C) as we observed in the pDC-antibody depletion experiments. While the reduction in IL-12p40 concentrations and cell numbers were not as great as seen in the Ab depletion model, these data suggest that the observations we made with the pDC depleting antibody can be reproduced in general with the DT model and that these results are indeed pDC specific. Additionally, the baseline level of inflammation in the BDCA2-DTRtg mice appeared to be greater than found in the Ab depletion model, thus presenting a possible explanation for the differences between the two models (Figures 1D and 4A).

### FLT3L-induced increase in pDCs leads to enhanced pulmonary inflammation in response to CP infection

**Figure 5:**
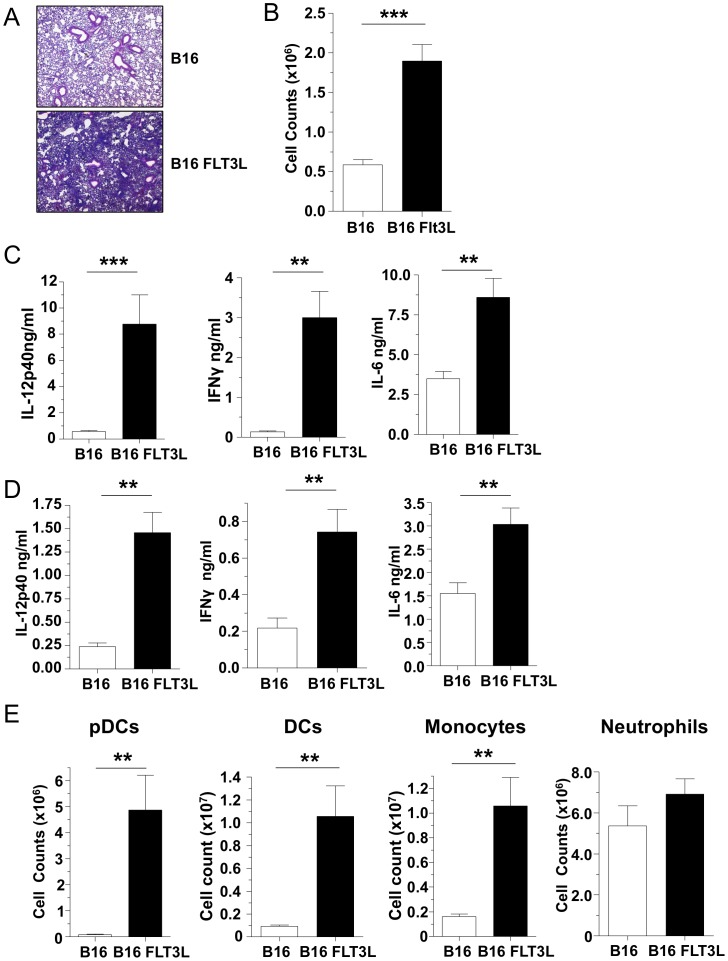
Mice with FLT3L-induced dendritic cells have increased proinflammatory responses to CP infection. (**A–E**) C57Bl/6 mice were injected 3×10^6^ FLT3L expressing cells (or control cells) s. c. 10 days prior to infection with 1×10^6^ IFU CP i. t. (n = 7–9). Mice were sacrificed 3 days after infection. (**A**) H&E staining of lungs (40x). (**B**) BALF cell count. (**C**) IL-12p40, IFN-γ, and IL-6 concentrations were measured in the BALF by ELISA. (**D**) IL-12p40, IFN-γ, and IL-6 concentrations were measured in lung homogenates by ELISA. (**E**) pDCs, cDCs, monocytes, and neutrophils were counted in lung single cell suspensions by Flow cytometry. Data for all experiments shown represent at least two independent experiments pooled together. *p<0.05, **p<0.01, ***p<0.001 (Student's *t* test used unless otherwise noted).

We next sought to assess whether the addition of pDCs to the system would results in the opposite effect; increasing inflammation in the lungs in response to CP infection. To do this, we used the FLT3L overexpressing cell transplant model where FLT3L expressing cells are injected s.c. into the mice to deliver large amounts of FLT3L, which leads to an increase in pDCs and cDCs in vivo [23]. Mice were injected with either FLT3L+ cells s.c. or control cells and the transplanted cells were allowed to grow for 10 days. Afterwards, the mice were infected with CP as before and sacrificed three days later. Mice that received FLT3L expressing cell transplantation showed a marked increase in inflammation in the lungs and BALF cell counts were significantly higher (Figure 5A and B). IL-12p40, IL-6, and IFN-γ concentrations were all higher in the FLT3L overexpression CP infected mice in both the BALF and lung homogenates (Figure 5C and D). Importantly, pDC numbers in the lung were significantly increased in the FLT3L treated mice compared to controls (Figure 5E). Additionally (and as expectedly), the number of monocytes and cDCs were also increased in the FLT3L treated mice (Figure 5E). However neutrophil numbers remained the same (Figure 5E). Since the mice were sacrificed on day 3 after infection, similar to our original experiment (Figure 1E), we did not see any difference in CP burden at this time (data not shown). Importantly, uninfected mice bearing control or FLT3L expressing cells did not shown any signs of overt inflammation (Figure S5A, B). pDCs, cDCs, and monocyte numbers were increased in FLT3L bearing mice as expected (Figure S5C–G). It should be noted that there were some increases in IL-12p40 levels in the FLT3L bearing mice, presumably due to the large influx of pDCs, cDCs, and monocytes (Figure S5H, I). However, IL-6 levels were unchanged and while there was an increase in IFNγ amounts in the lung homogenates of uninfected FLT3L bearing mice, the amount of IFNγ present was very low (<100 pg) (Figure S5H, I).

### pDCs are responsible for the increase in inflammation in FLT3L treated mice

**Figure 6:**
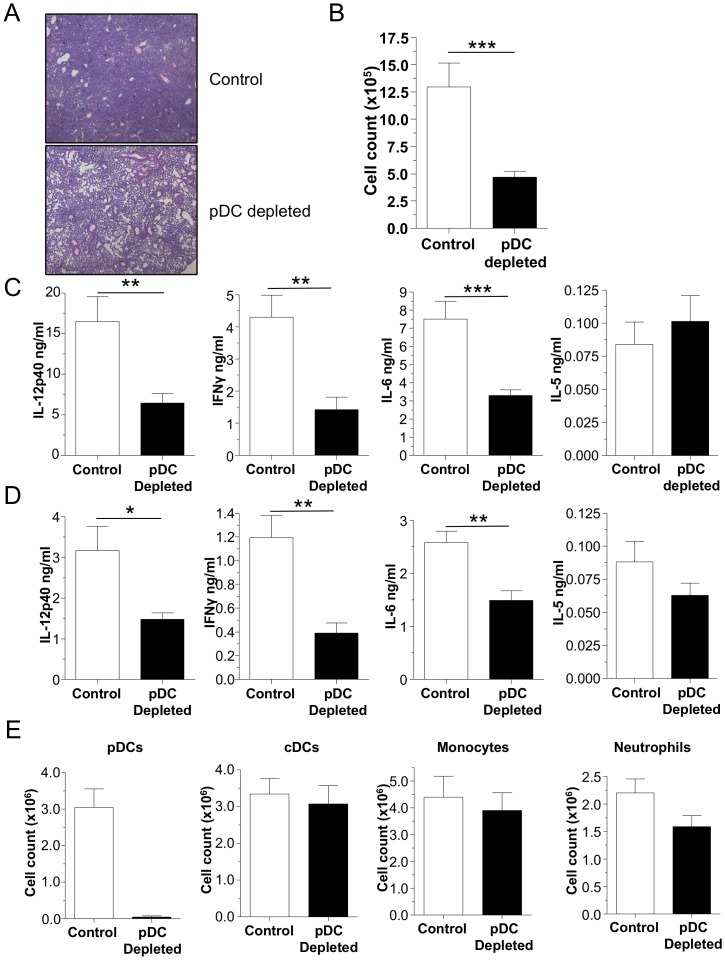
pDC depletion reduces inflammation in mice with FLT3L-induced dendritic cells during CP infection. (**A–E**) C57Bl/6 mice were injected 3×10^6^ FLT3L expressing cells s. c. 10 days prior to infection with 1×10^6^ IFU CP i. t. The mice were injected i. p. with either 500 µg mAb 927 or IgG control every other day during infection (n = 8–9). Mice were sacrificed 3 days after infection. (**A**) H&E staining of lungs (20x). (**B**) BALF cell count. (**C**) IL-12p40, IFN-γ, IL-6, and IL-5 concentrations were measured in the BALF by ELISA. (**D**) IL-12p40, IFN-γ, IL-6, and IL-5 concentrations were measured in lung homogenates by ELISA. (**E**) pDCs, cDCs, monocytes, and neutrophils were counted in lung single cell suspensions by Flow cytometry. Data for all experiments shown represent at least two independent experiments pooled together. *p<0.05, **p<0.01, ***p<0.001 (Student's *t* test used unless otherwise noted).

While greater amounts of cytokines were present in the lungs of FLT3L treated mice, we could not conclude that this observartion was strictly due to increased pDC numbers, as cDCs and monocytes were also increased. In order to address this question, we utilized the FLT3L overexpressing cell transplant model again, except we now depleted pDCs with antibody in one group. Depletion of pDCs in these FLT3L treated mice resulted in reduced inflammation and BALF cell counts 3 days after infection (Figure 6A and B), as well as a significant reduction in IL-12p40, IFN-γ, and IL-6 concentrations in both the BALF and lung homogenates after pDC depletion (Figure 6C and D). Importantly, pDC depletion was extremely effective despite the increased numbers of pDCs in the FLT3L treated animals (Figure 6E). While pDC numbers were down, there were no differences in the numbers of cDCs, monocytes, and neutrophils in the lungs of the pDC-depleted animals compared to controls (Figure 6E). These data strongly suggest that the reduction in BAL cells, inflammation, IL-12p40, IFN-γ, and IL-6 that was observed in pDC-depleted mice were indeed due to the specific depletion of pDCs during CP infection. Additionally, we found that IL-5 levels were unchanged in this model (Figure 6C, D) and was present in similar quantities compared to the amounts shown previously (Figure 2A, B).

### pDC depletion leads to a chronic lung inflammation with iBALT like structures

**Figure 7:**
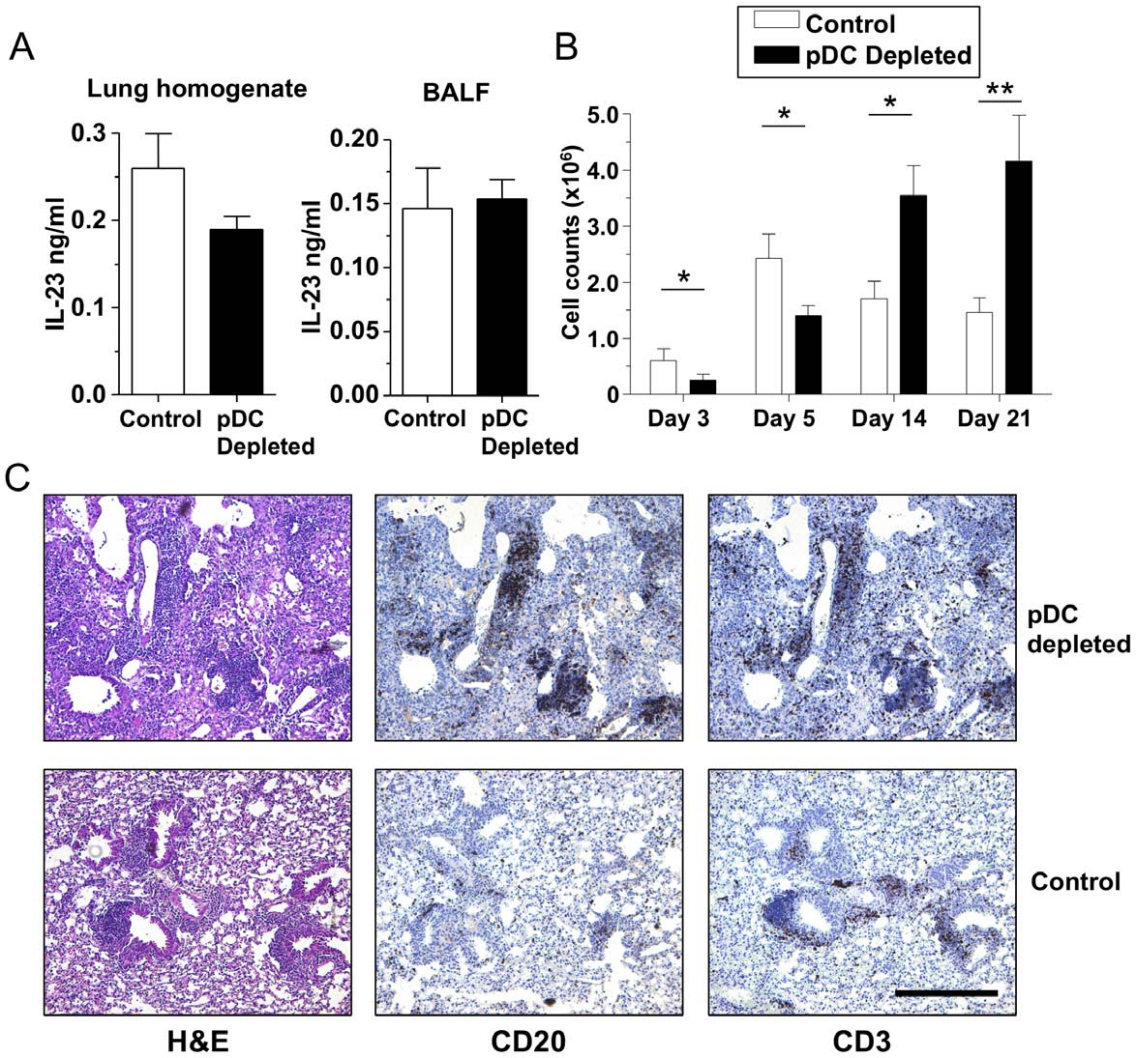
pDC depletion induces prolonged inflammation and iBALT like structures. (**A–C**) C57Bl/6 mice were infected with 1×10^6^ IFU CP i. t. and were injected i. p. with either 500 µg mAb 927 or IgG control (n = 7–10) every other day. Mice were sacrificed on days 3, 5, 14, and 21 after infection. (**A**) IL-23p19 was measured in the lung homogenates and BALF 21 days after CP infection. (**B**) CD8+ T-cells were counted in lung single cell suspensions by Flow cytometry. (**C**) Day 21 post infection lung sections stained for CD20 and CD3 in pDC-depleted and control mice. Images were taken at 100X. Scale bar indicates 0.25 µm. Data for all experiments shown represent at least two independent experiments (pooled together). *p<0.05, **p<0.01, ***p<0.001 (Student's *t* test used unless otherwise noted).

While pDC depletion during CP infection led to a reduced lung inflammation early on and delayed bacterial clearance, there was a significant increase in inflammation by day 21 (Figure 1C and E) and a significant increase in IL-12p40 concentrations in both the BALF and lung homogenates (Figure 2A and B), which was most likely due to the secondary effect of delayed bacterial clearance. While we did not detect any IL-17A in the lung homogenates during the acute phase of CP infection (data not shown), and the late increased lung inflammation was associated with elevated levels of IL-12p40, we measured IL-23 concentrations in the BALF and lung homogenates on day 21 as IL-12p40 is shared by both IL-12 and IL-23. However, we did not find any differences in IL-23 concentrations between pDC-depleted and control animals (Figure 7A) indicating that the increase in IL-12p40 concentrations is most likely due to increased IL-12. CD8+ T-cells in the lungs were also reduced early in the lung, followed by a significant increase in the numbers of CD8+ T-cells on days 14 and 21 (Figure 7B), coinciding with the increased IL-12 amounts and persistent inflammation. There was also a decrease in CD4+ T-cell numbers 5 days after infection, followed by an increase on days 14–21 (Figure S6A). Additionally there was an increase in CD4+ FoxP3+ Tregs on day 14 (Figure S6B). This late inflammation was further characterized by conducting a more in depth analysis of the histology. Looking at the H&E stainings of pDC-depleted day 21 lung samples, we noticed large groups of inflammatory cells surrounding airways and vasculature (Figure 7C). We stained for the presence of B (CD20) and T cells (CD3) and found a large presence of B and T cells in these cellular accumulations, similar to inducible bronchus associated lymphoid tissues (iBALT) (Figure 7C). Taken together these data indicate that the depletion of pDCs during CP infection in mice results in delayed bacterial clearance, and what appears to be a secondary and delayed lung inflammation characterized by increased IL-12, cellular infiltrates, and T-cells, as well as iBALT like structures that contain large numbers of B and T-cells.

## Discussion

Plasmacytoid dendritic cells are well known for their robust type I interferon response to viral infections, and for their ability to induce tolerance via Treg induction. In this study we sought to address a poorly understood facet of pDC biology; their role in immune responses to bacterial infections. Here we found that depletion of pDCs during a *Chlamydia pneumoniae* infection in mice resulted in delayed lung inflammation and bacterial clearance, with a significant reduction in early cytokine production. This resulted in an increase in a late chronic inflammation characterized by cellular infiltrates, iBALT like structures containing large numbers of B and T-cells, and increased IL-12.

One caveat to the antibody depletion model of pDCs (BST2 based) is the possible depletion of other activated immune cells [19]. However we have addressed the specificity issue by confirming our findings in the DT depletion model using the recently described BDCA2-DTRtg mice [22]. While the BDCA2-DTRtg mice had similar reductions in cytokine production and BALF cell counts compared with the Ab depleted mice, the changes in cell numbers were not as impressive. However, the BDCA2-DTRtg mice also appeared to have a higher baseline inflammation as assessed by BALF cell counts, thus making direct comparisons between the two models more difficult.

Very little is known about the role of pDCs during bacterial infections and their function in innate immune responses are just being investigated. In one study, pDCs were able to stimulate CD8 T-cell proliferation and IFN-γ production during a *Listeria monocytogenes* infection [24]. pDCs were also required during a CpG induced protective immune response to *Listeria* infection [25]. However, using a DTR ablation model, Takagi *et al* found that pDC depletion increased survival during *Listeria* infection by reducing overall inflammation [17]. Additionally, in a short study published by Ang *et al*, pDCs were induced during *Legionella pneumophila* infection and pDC depletion led to a delay in clearance of the bacteria, but its effect on inflammation was not described [16].

We had previously shown that the numbers of pDCs increased in the lungs of CP infected mice [10]. Similar to Ang *et al*, we found that pDC depletion resulted in a delayed bacterial clearance [16]. However, we also saw a dramatic decrease in inflammation early during lung infection with CP. While Takagi *et al* also found a decrease in inflammation during Listeria infection and pDC depletion [17], this was actually beneficial to the host. However, in our study, depletion of pDCs lead to delayed bacterial clearance and a prolonged chronic inflammation after CP infection. Thus depending on the type of bacterial infection, pDCs may play different roles and their depletion may result in beneficial or detrimental effects to the host.

Whether pDCs in the lung respond to CP infection directly, or by some secondary signal is currently unknown. CP infection is detected by both TLR2 and TLR4 [8], however, pDCs generally lack surface Toll-like receptors [26]. It is currently unknown if pDCs can sense CP via NOD/Rip2 signaling, nor has there been any studies indicating TLR9 detection of CP infection. We have found that bone marrow derived pDCs can make IL-12 in response to CP infection but the mechanism is not known (data not shown).

While pDCs can make IL-12 themselves, the significant reduction in IL-12p40 levels seen in pDC-depleted animals early during CP infection is unlikely due to any IL-12 the pDCs produced themselves as there are relatively low numbers of pDCs present in the lungs. Thus it is more likely that pDCs provide some other signal that induces macrophages and cDCs to produce larges amounts of IL-12. pDCs are known for their ability to make type I interferon, however, one study found that mice that lacked the type I IFN receptor, if anything, cleared CP infection better than WT mice [27]. Thus it is unlikely that our observations during pDC depletion are due to any involvement of type I IFN. pDCs are also known to influence Tregs via the production of indoleamine 2–3 dioxygenase (IDO) [13]. However, mice fed 1-methyltryptophan (an IDO inhibitor) during CP infection did not differ from control mice in their immune responses [28]. Another possible mechanism could be that pDCs are required for cross-presentation to naive CD8 T cells [29], [30]. Indeed we found a reduction in CD8 T cells early during CP infection in the lungs of pDC-depleted animals. However, there were increased CD8 T cells late during infection. Finally, pDCs have also been implicated in NK cell activation [31]–[33], a crucial source of IFN-γ early during CP infection. However, we did not find any significant changes in these cells during pDC depletion (data not shown).

Björck *et al* recently found that pDCs can come in two generic phenotypes, one that is proinflammatory, and another that is more suppressive [21]. In our study we found that while inflammation and cytokine production were reduced in the lungs of pDC-depleted CP infected mice, an opposite result was observed in the draining lymph node. Restimulation of DLN cells (5 days after infection) in pDC-depleted animals resulted in increased IFN-γ production, an apparent paradox given the reduced inflammation seen in the lungs. However, similar to what Björck *et al* found, lung pDCs and DLN pDCs had different levels of proinflammatory and suppressive surface markers during CP infection, coinciding with their respective phenotypes. This highlights the complexity of studying the role of specific cell types using a depletion model. Perhaps the late increase in CD4 and CD8 T-cells in the lung that we observed in pDC-depleted animals is due to enhanced T-cell activity in the DLN as a result of the depletion of suppressive pDCs in the DLN. These questions would be difficult to address, however, as the proinflammatory version of pDCs can switch to the suppressive phenotype [21].

Perturbation of early innate immune responses to CP lung infection tends to results in an initial delay in inflammation and bacterial clearing, followed by an increase in chronic inflammation later on via secondary pathways that is poorly understood [6], [7]. pDC depletion also resulted in an early delay in cellular recruitment and defective cytokine production with hindered bacterial clearance. This lead to a profound delayed and chronic inflammation, characterized by increased inflammatory cells (especially CD8 T-cells), high levels of IL-12, iBALT (B-cell and T-cell positive) like structures in the lungs of pDC-depleted CP infected animals. iBALTs are thought to provide an enhanced local immunity against the offending pathogen [34]. However, the origin and function of these CP infection-induced structures are currently not known and are under intense investigations. While we do not know by what mechanism the late inflammation is generated, it is likely that it is either due to the significant delay in bacterial clearance and secondary responses, or a dysregulation in immune responses due to prolonged pDC depletion. Considering the associations between CP infections and chronic airway diseases such as asthma and COPD, understanding the development and effects of this prolonged inflammation is a high priority. pDCs clearly play a role for proper host immune responses to CP infection (that does not require type I IFN), suggesting a much greater influence on innate immune responses to bacterial infections, however, the mechanisms for these interactions still needs to be established.

## Materials and Methods

### Ethics Statement

All animal experiments were performed according to the guidelines and approved protocol (IACUC Protocol #2097) of the Cedars-Sinai Medical Center Institutional Animal Care and Use Committee. Cedars-Sinai Medical Center is fully accredited by the Association for Assessment and Accreditation of Laboratory Animal Care (AAALAC International) and abides by all applicable laws governing the use of laboratory animals. Laboratory animals are maintained in accordance with the applicable portions of the Animal Welfare Act and the guidelines prescribed in the DHHS publication, Guide for the Care and Use of Laboratory Animals.

### Mice

Female C57BL/6 mice 8 to 12 weeks of age (Jackson Labs- Bar Harbor, ME) were used throughout the study and were housed under specific pathogen free conditions [4]. BDCA2-DTR mice (C57Bl/6 background) were acquired from Jackson Labs (Bar Harbor, ME).

### Infection with *C. pneumoniae*

Lung homogenates from *C. pneumoniae* (CM-1 (ATCC, Manassas, VA)) infected mice were propagated in HEp2 cells and counted as previously described [6]. HEp-2 cells and *C. pneumoniae* stocks (suspended in 0.2 M sucrose, 0.02 M sodium phosphate (pH 7.2), 5 mM_glutamate buffer) were determined to be free of Mycoplasma contamination by PCR [6]. CP was purified from Hep2 cell debris by centrifugation [6]. Mice were intratracheally infected with *C. pneumoniae* by inoculating with either 1×10^6^ or 3.5×10^6^ IFU in 60 µl PBS.

### pDC and cDC induction via FLT3L expressing cells

B16 and FLT3L overexpressing B16 cells were grown in RPMI at 37°C 5% CO_2_. 3×10^6^ cells were injected s. c. in the necks of the mice and allowed to grow for 10 days prior to infection studies [35], [36].

### Reagents

mAB 927 was purified for use by Harlan (USA) from a hybridoma. For pDC depletions, 500 µg mAB 927 (pDC depleting mAB 927 hybridoma was provided to us by Marco Colonna, Washington University, MO.)[19] was injected i. p. every other day. Rat IgG from serum was used as antibody control (Sigma, MO). In some experiments, 100 ng diphtheria toxin (Sigma, MO) was injected every other day i. p. to deplete pDCs in BDCA2-DTR mice. B16 and B16 FLT3L expressing cells were propagated in RPMI [23].

### Flow Cytometry

The lymphocytic makeup of the lungs after infection was analyzed by flow cytometry of lung homogenates. Briefly, lymphocytes were isolated by digesting the lung tissue at 37uC for 20 minutes in HANKS buffer containing Liberase (Roche Diagnostics, Indianap- olis, IN, USA) and 50 units/ml DNase I (Roche Diagnostics) and filtering through a 70 mm cell strainer (BD Biosciences). Erythrocytes were depleted by lysis buffer before staining. Isolated single cells were stained with following specific mAbs: CD16/32 (clone 93), GR-1 (clone RB6-8C5), CD11b (clone M1/70), F4/80 (clone BM8), CD11c (clone N418), CD4 (clone RM4-5), CD8 (clone 53-6.7), B220 (clone RA3-6B2), CD3 (clone 145-2C11), CD19 (clone eBio1D3), CD80 (clone 16-10A1), Siglec-H (clone ebio440c), MHCII (clone M5/114.15.2), ICOSL (clone HK5.3) and FoxP3 (Clone FJK-16S) were purchased from eBioscience as direct conjugates to either FITC, PE or PerCP-Cy5.5, APC, biotin, or E450. Anti-BST2 (120G8/04) directly conjugated to APC was purchased from Imgenex. Anti-CD9 (clone KMC8)-biotin and anti-CD40L (clone 39H5) FITC were purchased from Beckton Dickenson and ABD Serotec respectively. Cells were identified based on expression of following antigens: alveolar macrophages (F4/80+ and CD11c+), DC (F4/80- and CD11c+/CD11b+), Neutrophils (GR-1+ and CD11b+), T cells (CD3+, CD4+, CD8+ and FoxP3+), NK cells (NK1.1+), B cells (B220+ and CD19+), monocytes (GR-1+, CD11b+, SS low), and pDCs (B220+, BST2+, CD3/CD11b/CD19-). For intracellular IL-12p40 staining, cells were treated with Golgistop for 6 hours, then permeabilized using Cytofix/Cytoperm kit (BD Biosciences) and stained with conjugated anti-mouse IL-12p40 mAb (clone C17.8, eBiosciences). Flow cytometric analysis was performed using a CyAnTM flow cytometer (Beckman Coulter) and the data was analyzed using Summit (Dako, Carpinteria, CA, USA) software.

### Histopathological analysis

Lungs were fixed in formalin buffer, paraffin-embedded, and hematoxylin and eosin (H&E)-stained sections were scored by a trained pathologist blinded to the genotypes as previously described [7]. Immunohistochemistry was performed using anti CD3 (clone 2GV6) prediluted from Ventana Medical Systems (Yuscon, AZ) and anti CD20 (goat polyclonal, 1∶50) from Santa Cruz Biotechnologies (CA).

### Detection of cytokines

The cytokine concentrations in the BALF and lung homogenates were determined using by OptiEIA Mouse IL-6, Mouse IL-5, and MCP-1 ELISA Set (BD Biosciences, San Jose, CA, USA) and Mouse IFN-γ, Mouse IL-12p40, Mouse IL-23, and Mouse IL-17A ELISA kit (eBioscience, San Diego, CA). The assays were performed as described in manufacturers' protocols.

### Ex vivo restimulation experiments

Single cell suspensions were isolated from the mediastinal lymph node and plated 2×10^5^ per well in a 96 well plate. Cells were stimulated for 72 h with either CD3/CD28 (1 µg/ml) or UVCP at an M.O.I. of 10. Supernatants were collected and assessed for cytokine production by ELISA.

### Statistics

Data are reported as mean values±SEM. Statistical significance was evaluated by Student's t test (two-tailed). In experiment where data was not normally distributed, the Mann-Whitney test was performed. In the case of survival study, statistical significance was evaluated by Fisher's exact test.

## Supporting Information

Figure S1 **pDC depletion using mAb 297.** C57Bl/6 mice were infected with 1×10^6^ IFU CP i. t. and were injected i. p. with either 500 µg mAb 297 or IgG control (n = 7–10) every other day. Mice were sacrificed on days 3, 5, 14, and 21 after infection. (**A**) pDCs were counted in lung single cell suspensions by Flow cytometry. (**B**) Representative scatter plot of pDCs in control and pDC depleted animals. Data for all experiments shown represent at least two independent experiments. *p<0.05, **p<0.01, ***p<0.001 (Student's t test).
(TIF)

Figure S2 **pDC depletion in uninfected WT mice.** C57Bl/6 mice depleted of pDCs for three days and sacrificed (n = 5). (**A**) H&E stained lung section of uninfected WT mice with and without pDC depletion (3 days). (**B**) BALF cell counts of uninfected WT mice with and without pDC depletion (n = 5) (3 days). Mice were assessed for IL-12p40 and IL-6 in the BALF (**C**) and lung homogenates (**D**), as well as single cell counts in the lungs (**E**). *p<0.05, **p<0.01, ***p<0.001 (Mann-Whitney).
(TIF)

Figure S3 **Cell counts in the draining lymph node during CP infection.** (**A-F**) C57Bl/6 mice were infected with 1×10^6^ IFU CP i. t. and were injected i. p. with either 500 µg mAb 297 or IgG control (n = 5) every other day. Mice were sacrificed 5 days after infection. Mediastinal lymph nodes were harvested and processed into single cell suspensions and analyzed by Flow cytometry. (**A**) Total cell counts. (**B**) cDC cell counts. (**C**) CD4+ Tcell counts. (**D**) CD8+ Tcell counts. (**E**) Treg cell counts. (**F**) CD19+ cell counts. Representative scatter plots are shown. Data for all experiments shown represent at least two independent experiments. *p<0.05, **p<0.01, ***p<0.001 (Mann-Whitney).
(TIF)

Figure S4 **pDC depletion using the diphtheria toxin ablation model.** BDCA2-DTRtg mice were infected with 1×10^6^ IFU CP i. t. and were injected i. p. with either 100 ng DT or PBS control (n = 5) every other day. Mice were sacrificed 3 days after infection. (**A**) pDCs were counted in lung single cell suspensions by FLOW cytometry. Data for all experiments shown represent at least two independent experiments. *p<0.05, **p<0.01, ***p<0.001 (Mann-Whitney).
(TIF)

Figure S5 **Mice with FLT3L induced dendritic cells have increased immune cell infiltrates but no inflammation.** C57Bl/6 mice were injected 3×10^6^ FLT3L expressing cells (or control cells) s. c. and sacrificed 13 (10+3) days after injection (n = 5). (**A**) H&E stained lung sections (**B**) BALF cell counts. (**C–G**) pDC, Alveolar macrophage, cDC, monocyte, and neutrophil cell counts were measured in lung single cell suspensions by FLOW cytometry. (**H**) IL-12P40, IL-6, and IFNγ amounts in the BALF and (**I**) lung homogenates. *p<0.05, **p<0.01, ***p<0.001 (Mann-Whitney).
(TIF)

Figure S6 **T-cells in the lungs during pDC depletion and CP infection.** (**A-B**) C57Bl/6 mice were infected with 1×10^6^ IFU CP i. t. and were injected i. p. with either 500 µg mAb 297 or IgG control (n = 7–10) every other day. Mice were sacrificed on days 3, 5, 14, and 21 after infection. (**A**) CD4+ T-cell counts were measured in lung single cell suspensions by Flow cytometry. (**B**) Treg T-cell counts and percentages were measured in lung single cell suspensions by Flow cytometry. Data for all experiments shown represent at least two independent experiments (pooled together). *p<0.05, **p<0.01, ***p<0.001 (Student's t test used unless otherwise noted).
(TIF)
