# Supplementary figures and images for: Plasmacytoid Dendritic Cells Play a Role for Effective Innate Immune Responses during Chlamydia pneumoniae Infection in Mice
Source: PLoS One. 2012 Oct 31;7(10):e48655. doi: 10.1371/journal.pone.0048655 (PMC3485374; doi:10.1371/journal.pone.0048655)

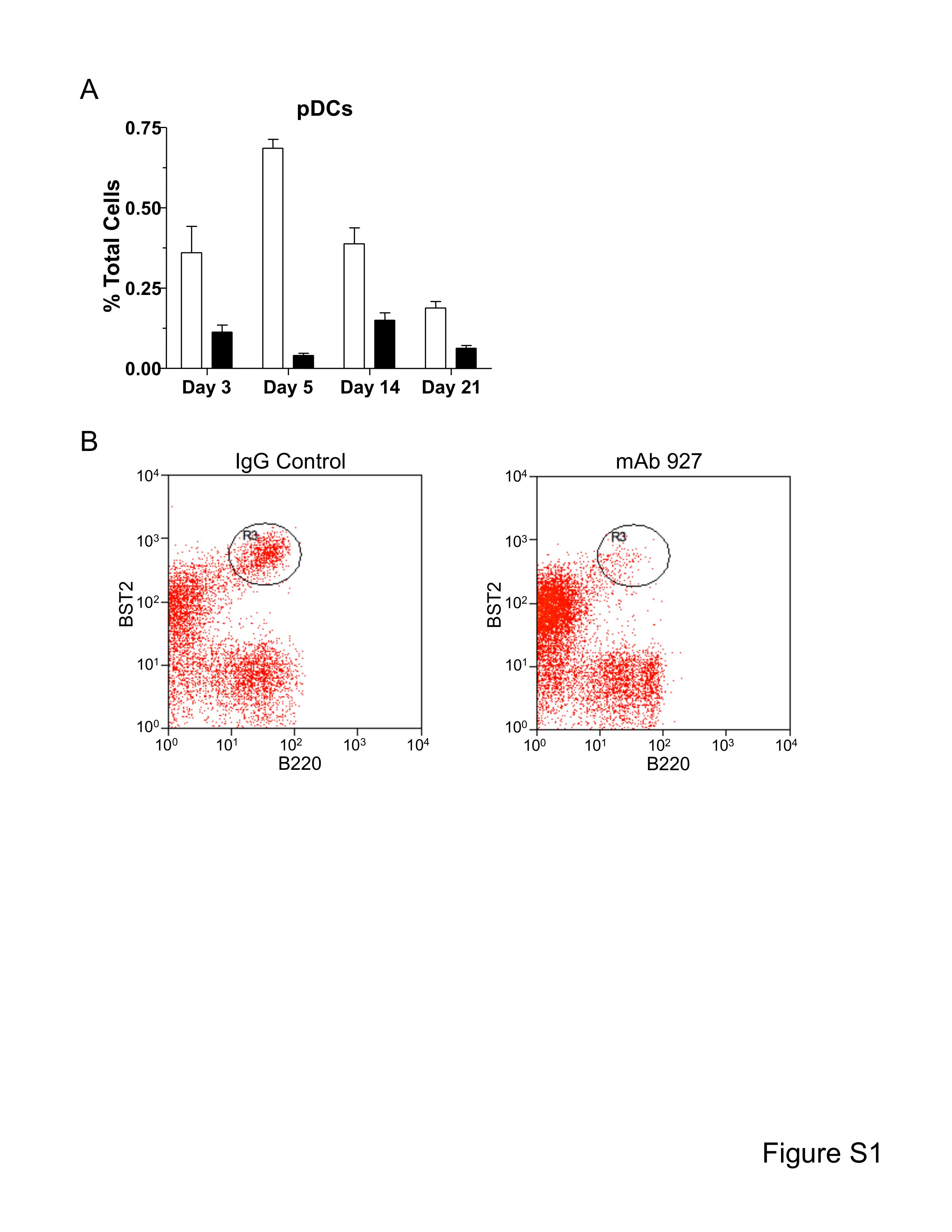

Supplement: Figure S1 — pDC depletion using mAb 297. C57Bl/6 mice were infected with 1×10^6^ IFU CP i. t. and were injected i. p. with either 500 µg mAb 297 or IgG control (n = 7–10) every other day. Mice were sacrificed on days 3, 5, 14, and 21 after infection. (A) pDCs were counted in lung single cell suspensions by Flow cytometry. (B) Representative scatter plot of pDCs in control and pDC depleted animals. Data for all experiments shown represent at least two independent experiments. *p<0.05, **p<0.01, ***p<0.001 (Student's t test). (TIF) [file pone.0048655.s001.tif]

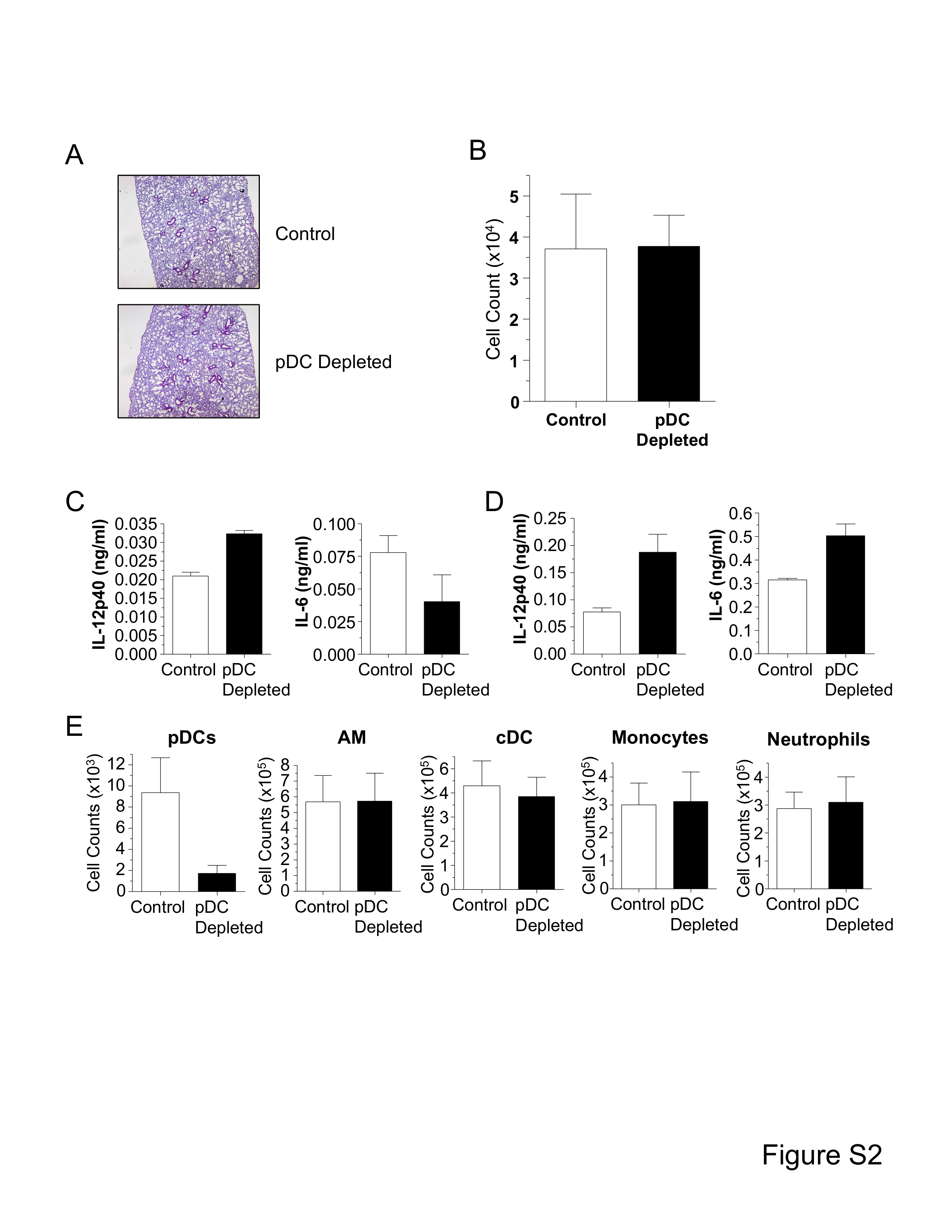

Supplement: Figure S2 — pDC depletion in uninfected WT mice. C57Bl/6 mice depleted of pDCs for three days and sacrificed (n = 5). (A) H&E stained lung section of uninfected WT mice with and without pDC depletion (3 days). (B) BALF cell counts of uninfected WT mice with and without pDC depletion (n = 5) (3 days). Mice were assessed for IL-12p40 and IL-6 in the BALF (C) and lung homogenates (D), as well as single cell counts in the lungs (E). *p<0.05, **p<0.01, ***p<0.001 (Mann-Whitney). (TIF) [file pone.0048655.s002.tif]

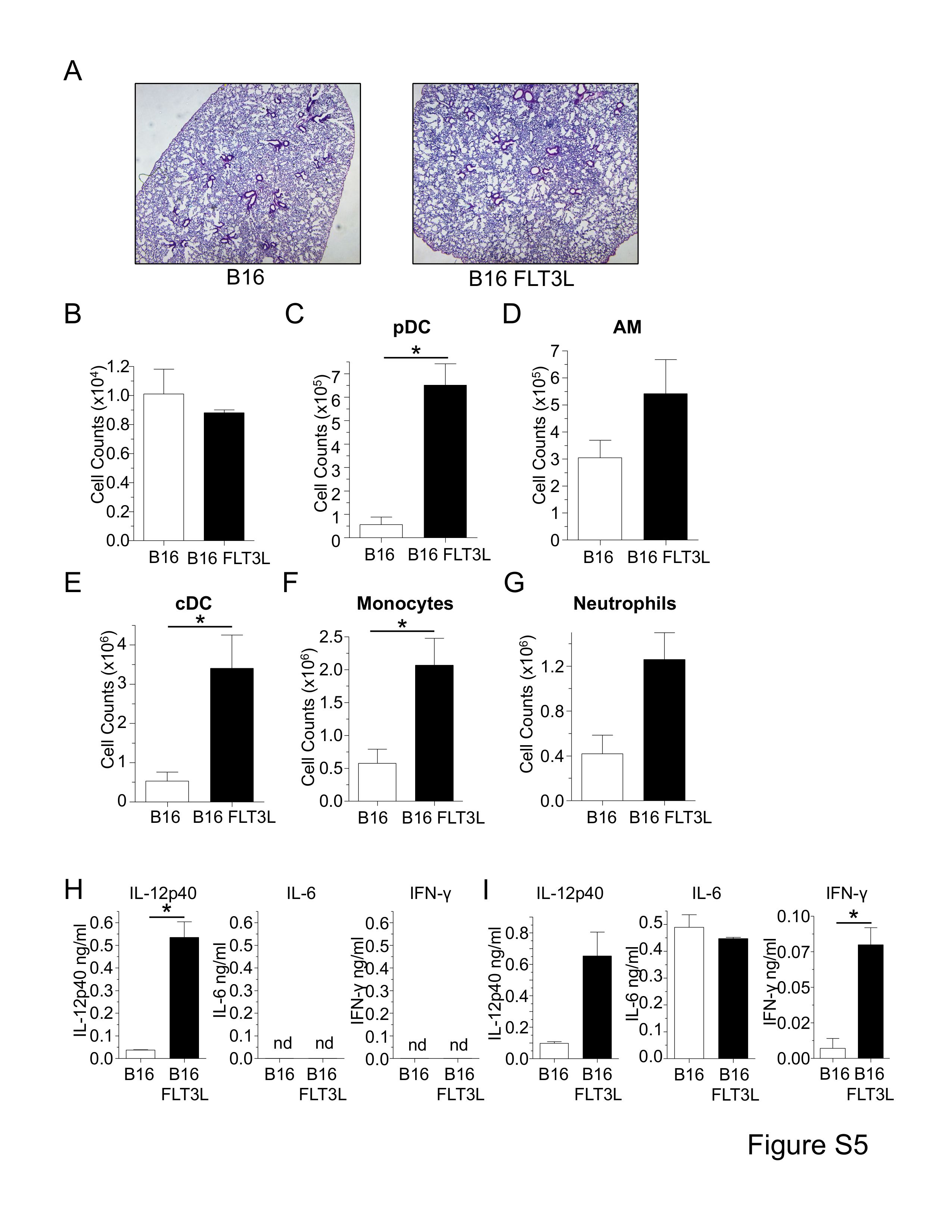

Supplement: Figure S5 — Mice with FLT3L induced dendritic cells have increased immune cell infiltrates but no inflammation. C57Bl/6 mice were injected 3×10^6^ FLT3L expressing cells (or control cells) s. c. and sacrificed 13 (10+3) days after injection (n = 5). (A) H&E stained lung sections (B) BALF cell counts. (C–G) pDC, Alveolar macrophage, cDC, monocyte, and neutrophil cell counts were measured in lung single cell suspensions by FLOW cytometry. (H) IL-12P40, IL-6, and IFNγ amounts in the BALF and (I) lung homogenates. *p<0.05, **p<0.01, ***p<0.001 (Mann-Whitney). (TIF) [file pone.0048655.s005.tif]
